# Supplementary material for: Patterns of Intron Gain and Loss in Fungi
Source: PLoS Biol. 2004 Nov 30;2(12):e422. doi: 10.1371/journal.pbio.0020422 (PMC532390; doi:10.1371/journal.pbio.0020422)
Supplement: Table S1 — Also available at http://genes.mit.edu/NielsenEtAl/. (4.3 MB ZIP). [file pbio.0020422.st001.zip › NielsenEtAl/html/29.html]

AN4201.1.NCU00129.1.MG10188.1.FG07277.1


```
 CLUSTAL W (1.82) Multiple Sequence Alignments - Introns Inserted


Sequence 1: NCU00129.1	573 aa
Sequence 2: FG07277.1	588 aa
Sequence 3: MG10188.1	587 aa
Sequence 4: AN4201.1	524 aa
Alignment Length: 610 aa
Number Identitical Residues: 312 aa
Alignment Score (without introns) 15324


MG10188.1 	MSGPPSRLKAVLGHLGLANAGAEATQGTGRPNHLHALSPTLFLQRAAAIEPEAEAVVHVT
NCU00129.1	MSGAPSRLRSVLNHLLPHGQ---------PAHHIHNLTPTVFLERAAAIEPEAEAIFHIT
FG07277.1 	MSDPPSRIRSILSHLRPHPA---------APVNYHTLSPTTFLERAAAIEPDAEAIFHIT
AN4201.1  	-------------------------------------------------MRKAEAIYHIT
          	                                                   .***: *:*

MG10188.1 	ANGRTLRRSYAELADRARGLAYYLAKKGFFRVGILAPNTPAFLESIYGVNASGGVIVP~V
NCU00129.1	ANGKTLRRSYIEFADRARGLAYYLRKHGYKRVGLLAPNTPAFLESVYGIVAAGGVIVP~V
FG07277.1 	VNGAVLRRSYAEFADRARGLAYYLLKHGYRRVGVLAPNTPAFLESIYGIVAAGAVIVP~A
AN4201.1  	ANNQVLRRTYQETADRARGLAYFLKKHGYKRVGILCPNTPAFLESIFGIAAAGAVNVS1V
          	.*. .***:* * *********:* *:*: ***:*.*********::*: *:*.* *. .

MG10188.1 	NYRLKQEDIAYIFEFANVDAIIVDAEFVGLLELYQKDHVNVPLIIDT0------------
NCU00129.1	NIRLKPEDITYIFDFAEVDSIIADAEYAHLLNSYKKEHPGVPVIIDV0------------
FG07277.1 	NYRLKQEDIAYIFDFAEVDCIIVDNEFVGLLDAYKEKHENVPLIVDM~VDFLPHEQAEAC
AN4201.1  	NYRLKEDDIAYIFTHSDAEIIIADKEYLPLLSAYRAAKPHVRVILDT~------------
          	* *** :**:*** .::.: **.* *:  **. *:  :  * :*:*              

MG10188.1 	----------DTDATEGELSGPFDECIAQGLQHDREAGGKGWDGLCQQPND-EDDIIAIP
NCU00129.1	----------DTDATEGALCGPFDEAVMEGLNHDKATGSKGWADLQNQTVANEDDMIAIP
FG07277.1 	FKAVANLAYQDNDATVGQLSGPFDDAVLEGLKHDIAQGSQGWAGLHSQARS-EDDMLAIP
AN4201.1  	----------DTDATEGQLAGPFDEAILEGLNYDRDTGRKGWSALESQAAS-EDDVIALA
          	          *.*** * *.****:.: :**::*   * :**  * .*.   ***::*:.

MG10188.1 	FTSGTTSKPKGVEYTHRGAYLAALANIVESGLNYNVGRCKYLWTLPM2FHAVG~WTFPWA
NCU00129.1	FTSGTTSKPKGCVYTHRGAYLATLANVIESGLNVADGRSKYLWTLPM~FHAMG~WTFPWA
FG07277.1 	FTSGTTSKPKGVVYTHRGSYLAAMANIVESGLNIG--RCKYLWTLPM2FHAVG~WTFPWA
AN4201.1  	YTSGTTARPKGVEYTHRGCYLAAMGNIIESGLNFQGRRCKYLWTLPM~FHAMG1WTFPWA
          	:*****::***  *****.***::.*::*****    *.******** ***:* ******

MG10188.1 	VTA1VRGTHVCLRKIDYPLIWRLLTGERVTHFNAAPTVCTLLCAADEAQRLPDPPVRVTV
NCU00129.1	VCA~VRGTHVCLRKIDYPLIWKLLKEEGVTHFCAAPTVNTLLCAAKEAEALP-KPVRVTV
FG07277.1 	VVA~VRGTNVCLRKIDYPLIWKLLKEEGITHFNAAPTVNTLLVAAKEAEKLP-HEVKVTV
AN4201.1  	VTA~VRGTHYCLRKIDYPQIWKLLKQEGVTHFNAAPTVNTLLCSSPEAEPLP-EPVHVTV
          	* * ****: ******** **:**. * :*** ***** *** :: **: **   *:***

MG10188.1 	AASPPTAHLFRQM~TELNLSPVHVYGMTE~TYGPITKGYHMPAWETLPEADKFARMARQG
NCU00129.1	AASPPTPHLFEQM~TSLNLFPVHVYGLTE~TYGPITRGYILPSWDNLPPHDKYAKMARQG
FG07277.1 	AASPPTGYLFEQM0TKLNLIPVHVYGMTE1TYGPSTKCYTLPEWDSLPPTEKYAKMSRQG
AN4201.1  	AASPPTPHLFEQM~TGLNLHPVHVYGMTE~TYGPITKGYYMTAWENLPRKEKFARMARQG
          	****** :**.** * *** ******:** **** *: * :. *:.**  :*:*:*:***

MG10188.1 	HGFLTSLPIRIVKPEEAENGVLIDVAKDGVEIGEIVFAGNICAKGYYNDPEATKKMFAGG
NCU00129.1	HGFITSLPARIIKPDQPE-GVLIDVEKNGQEVGEIIFTGNICCKGYYKDPEATRKLFAGG
FG07277.1 	HGFITSLPIRIIKPDQPQ-GVLIDVAKDGKEIGEIVFMGNICAKEYYKDPAATRELFAGG
AN4201.1  	HGFLTSLPVRVIQTEVPE-GTIIDVKQDGKEIGEIVFVGNICARGYYKDPEATRKLFAGG
          	***:**** *:::.: .: *.:*** ::* *:***:* ****.: **:** **:::****

MG10188.1 	VLHSGDLAVWHPDGSAQILDRQKDIIISG1GENISSVALESMLVQHPDVLEAGVVAVPDS
NCU00129.1	MLHTGDLAVWHPDGSIHIQDRAKDIIISG1GENISSVALESMLAEHPDILEAGVVAVPDS
FG07277.1 	VLHSGDLAVWHPDGSAQIQDRAKDIIISG~GENISSVALESMLAQHPDILEAGAVAVPDS
AN4201.1  	VLHSGDLAVWHPDGAIQILDRAKDIIISG1GENISSVALESMLVTHPDILEAGVVAVPDS
          	:**:**********: :* ** ******* *************. ***:****.******

MG10188.1 	HWGERPKAYVTVRRSKEAGMEPLTGQGLIDWAKHQSAISKFMIPREVEIVDELPKTSTGK
NCU00129.1	HWGERPKAYITLKEGRET---SLTGQDVIDWAKHNSSISKFMVPREVEIVKELPKTSTGK
FG07277.1 	HWGERPKVYVTVKDGK-----SVTGDEIIQWAKHQSDISRFMVPREVEIVDELPKTSTGK
AN4201.1  	HWGERPKAFVTVKPGR-----QLKGKDVVDWARNTSGISKFMVPREVEVVAELPKTSTGK
          	*******.::*:: .:      :.*. :::**:: * **:**:*****:* *********

MG10188.1 	IKKNELR~EWAKHGKSSLE
NCU00129.1	IKKNELR~VWAKTGTIPN-
FG07277.1 	IKKNVLR~EWAKHGRT---
AN4201.1  	LRKNVLL1WLDR-------
          	::** *     :
```
